# Phospholipase C signaling activated by parathyroid hormone mediates the rapid osteoclastogenesis in the fracture healing of orchiectomized mice

**DOI:** 10.1186/s12891-018-2231-3

**Published:** 2018-08-29

**Authors:** Wei Li, Liang Yuan, Guojun Tong, Youhua He, Yue Meng, Song Hao, Jianting Chen, Jun Guo, Richard Bringhurst, Dehong Yang

**Affiliations:** 1 Department of Spinal Surgery, Nanfang Hospital, Southern Medical University, Guangzhou, 510515 China; 2 Endocrine Unit, Massachusetts General Hospital, Boston, MA 02114 USA

**Keywords:** Parathyroid hormone, Fracture healing, Phopholipase C, Osteoporosis, Osteoclastogenesis

## Abstract

**Background:**

The age-related osteoporosis is an increasing risk severely threatening the live quality of aged people. Human parathyroid hormone (hPTH) is applied to the therapy of osteoporosis successfully, however, the mechanism, especially the signaling pathway activated in the healing fracture by PTH is still unknown.

**Methods:**

The once daily injections of hPTH(1–34) and GR (1–34) (the PLC deficient analog) into the orchiectomized male mice with bone fracture, were started at the second day after fracture and lasted for 4 weeks. To explore the role of phospholipase C signaling in the androgen-deficient fracture healing, the fracture healing were evaluated via radiography, micro-CT, biomechanics testing, serum biochemistry, bone marrow cell culture and gene expression quantification.

**Results:**

After two weeks of fracture, both peptides significantly increased bone mineral density (BMD), bone mass content (BMC) and bone volume (BV/TV) in the healing area. However, compared to hPTH(1–34), GR(1–34) induced more woven bones, the higher BMC and BMD, as well as the less serum TRAP and osteoclasts. After four weeks of treatment, the effects of hPTH(1–34) on fracture healing showed no difference to those of GR(1–34). Consistently, GR(1–34) induced the similar osteogenesis but less osteoclastogenesis under the ex vivo condition immediately after administration compared to hPTH(1–34), which was verified by the weaker activation of RANKL, NFATC1, TRAP and Cathepsin K in GR(1–34) treatment.

**Conclusion:**

These results indicated that the PLC signaling activated by the intermittent injection of hPTH(1–34) leads to the bone resorption by rapidly activating the osteoclastogenesis in the fracture healing zone.

**Electronic supplementary material:**

The online version of this article (10.1186/s12891-018-2231-3) contains supplementary material, which is available to authorized users.

## Background

Previous studies showed that both the osteoporosis-related morbidity and mortality in men actually were higher than those in women in later life [1, 2]. The increased risk of fractures is closely associated with the decline of testosterone production, which causes a high turnover bone loss from cancellous bone sites [1, 3, 4]. However, supplement of androgen or testosterone replacement therapy takes a high risk of causing complications in circulating, urinary and endocrine systems [5–7]. By far, the most reliable bone-forming medicine for male osteoporosis without triggering severe complications is human parathyroid hormone (hPTH) [2], a key factor regulating the systemic metabolism of calcium and phosphate [8]. Animal studies demonstrate that hPTH promotes bone volume [9], fracture healing [10–12] and spinal bone fusion [13]. In practice, the intermittently subcutaneous injection of hPTH successfully ameliorates the osteoporosis and osteoporotic fracture in human [14–18]. Although PTH is secreted as a peptide with 84 amino acids, the 34 amino acids at the amino-terminus, namely the PTH(1–34) possesses the entire activity of the full length PTH [19], which has been proved in the animal fracture models of tibial and other bones [20, 21]. However, if the effect of PTH on the entire bone is identical or different to that on the fracture site is still in debated, because the intermittently subcutaneous injection of hPTH (1–34) enhances the bone mineral density (BMD) and bone formation in callus, as well as several osteogenic markers in serum [22–25]. In contrast, the prolonged exposure to PTH leads to bone resorption, as opposed of bone formation in the intermittent exposure [19, 26].

Both PTH and PTH(1–34) work through the type I PTH receptor (PTHR1) to activate several G protein coupled signaling cascades, including cyclic adenosine monophosphate (cAMP)/protein kinase A (PKA), phospholipase C (PLC)/protein kinase C (PKC) and PLC-independent/PKC [19, 26, 27]. Since the activation of each pathway has been mapped to the different domains within hPTH(1–34), the ligand analogs of hPTH(1–34) specific for a certain pathway were developed by mutating the key amino acid residues within hPTH(1–34) [19], which provides a powerful tool to analysis the functional of a specific signaling during bone formation and resorption. Combining these analogs with the genetically modified mice, the effects of the different signaling pathways induced by hPTH on osteoblastogenesis, osteoclastogenesis and bone metabolism have been widely investigated [9, 28, 29]. The analog of hPTH(1–34) which combines the mutations of Ser^1^- > Gly^1^ and Glu^19^- > Arg^19^ into hPTH (1–34), namely the GR(1–34), is able to accumulate cAMP as hPTH(1–34) does, but unable to activate PLC even at a high concentration [26].

As reported previously, the cAMP/PKA mediates the beneficial effects of hPTH on fracture healing [9]. Our recent study and recent unpublished data revealed that the PKA-dependent PKC contributed to the effects of cAMP/PKA on bone formation [27], and the 29–34 amino acids of hPTH [hPTH(29–34)] triggers PLC-independent/PKC signaling [26]. These findings indicate that the PLC signaling associated with PTH are much more compound than expected. Thus, to clarify if the PLC signaling involved in the controversial functions of PTH administration would shed novel light on bone metabolism and osteoporosis treatment. In this study, the orchiectomized (ORX) male mice were employed as the model of androgen-related osteoporosis. By comparing the effects between the intermittently administrated hPTH(1–34) and GR(1–34), we explore that the role of PLC signaling performed in the fracture site of the ORX male mice.

## Methods

### Animals

One hundred and forty-seven C57BL/6 J male mice of seven-week-old were purchased from and acclimatized for 1 week before orchiectomy at the Laboratory Animal Center of Southern Medical University (Guangzhou, China). The animal research protocol was approved by the Animal Care and Use Committee of Southern Medical University. All applicable Southern Medical University guidelines for the care and use of animals were followed. All procedures performed in studies involving animals were in accordance with the ethical standards of the Southern Medical University.

### Orchiectomy and bone fracture model

Mice were anesthetized using the MATRX VMR small animal anesthesia machine (model VMR; USA) with continuous inhalation of 2% isoflurane mixed with oxygen. The sham treated animals underwent skin and scrotum incision without the testis removal, while the ORX mice had both testes entirely removed as previously described [2]. One week after surgery, a cross-sectional fracture was generated in the mid-shaft of the right femur by inserting a pin (0.45 mm in diameter) into the marrow cavity from the distal end and then, penetrating through the mid shaft of the femur. An incision was made in the middle region of the right thigh to expose the mid-shaft of the femur into the intermuscle space. A complete fracture was made by cutting the shaft of the femur, where the intramedullary pin was remained to stabilize the fracture ends [12].

### Administration of hPTH(1–34) and the analog GR(1–34)

hPTH(1–34) and GR(1–34) were synthesized at GL Biochem (Shanghai, China). The preparation for administration followed the protocol as described before [9]. Both peptides were injected at the 40 μg/kg subcutaneously once daily for 5 days in every week [30]. The same volume of vehicle (0.05 ml) was administrated into control group. The body weight, serum testosterone, and the bone quantity and quality in the fracture area were measured after the 2 and 4 weeks of injection.

### Biochemical assays

Half of one milliliter of blood was collected from the animals after general anesthesia, placed at room temperature for 15 min and then, centrifuged at 4000 rpm to obtain the serum. Enzyme-linked immunosorbent assay (ELISA) kits were applied to detect the levels of serum testosterone (Boster Biological Technology CO. Ltd., Wuhan, China), tartrated-resistant acid phsphatase (TRAP) (Cusabio, Wuhan, China), N-terminal propeptide of type I collagen (P1NP) and C-terminal collagen-type I fragments (CTX) (Immunodiagnostic Systems, Fountain Hills, AZ). Alkaline phosphatase (ALP) was detected with a colorimetric kinetic determination kit by following the manufacturer’s instructions (Byotime, Beijing, China).

### Measurement for bone mineral densitometry and bone mineral content

After the mice were sacrificed by carbon dioxide anesthesia and cervical dislocation, the right femur containing the growth of the callus was fixed in 4% paraformaldehyde for 48 h. Then, the soft tissues and intramedullary pin were removed. Bone mineral density (BMD) and bone mineral content (BMC) at the callus were measured by dual-energy X-ray absorptiometry (DEXA) with a densitometer (XR-36, NORLAND Inc., WI, USA). The region of interest (ROI) was a rectangular area (5.4 mm × 3.5 mm) centering on the fracture line, which contained both the newly generated callus tissues and the original bone.

### Micro-computed tomography

Micro-Computed tomography (Micro-CT) analysis was conducted by the μCT80 (SCANCO MEDICAL Inc., Switzerland) with the software μCT Evaluation Program V6.5–1 in the specimens after DEXA measurements. Image recording was confined to the callus of the fractured femur. Images of the femur mid-shaft (5.40 mm in length centering on fracture line) were performed using an isotropic voxel (12 μm in size). The grayscale threshold was set up at 220, meaning the values of mineralized tissue are greater than 220. The ROI of measurements was localized to a cylindrical space (5.4 mm in height and 3.5 mm in diameter) focusing at the middle point of fracture line. Three-dimensional (3D) pictures were also reconstructed based on the images from the three spatial dimensions to show the fracture healing. Samples from 12 mice from each group underwent micro-CT scanning and half of these samples were then tested for the biomechanical properties.

### Biomechanics testing

Six mice from each group were sacrificed by carbon dioxide anesthesia and cervical dislocation for biomechanics testing. The bone was immersed in PBS for 30 min after the removal of intramedullary pin and surrounding soft tissues, and then, placed between two plates (a span of 8 mm) with the medial and anterior sides facing forward and down, respectively. The bending rigidity of the healing femur was measured on the fourth week after injection by a three-point bending procedure using an Electropuls Test System (E1000; Instron, Inc., Illinois, USA). A central load was applied at the mid-callus with a constant rate of 2 mm/min until failure occurred. The rigidity of the femur was determined from the curve determined by the maximal force and displacement applied on the tested bone.

### Histology and histomorphometry

Collected femurs were decalcified in 20% ethylenediaminetetraacetic acid (EDTA) solution for 4 weeks, dehydrated, embedded in paraffin and then, sectioned in 5um for hematoxylin and eosin (HE) staining, Masson’s Trichrome staining (Maixin Biotech. Co. Ltd., Fujian, China), TRAP staining (Sigma-Aldrich, 387A-kit, USA), or TRAP immunohistochemical staining (Biosynthesis Biotechnology Co. Ltd., Beijing, China) with the standard protocols or the manufacturer’s instructions. 24 images (2 fields per section, 2 sections per sample; for callus formation analysis, magnification is 10; for osteoclasts analysis, magnification is 200) were photographed from 6 mice in each group and analyzed with Image J analysis software. ROI was defined as a rectangular box in the center of healing zone, in which the callus, woven bone and osteoclasts were outlined manually according to the specially stained color with the software.

### Ex vivo culture of bone marrow cells

The eight week old male mice were orchiectomized as described above. After one week of the orchiectomy, the mice were sacrificed by carbon dioxide anesthesia and cervical dislocation for femurs dissection and bone marrow collection. The isolated cells were seeded into 24-well plates precoated with collagen type I at 1 × 10^5^ cells/well for the 24 h culture, and then, induced for differentiation as described in the following. The osteogenic medium contained α-MEM supplemented with 10% FBS, 100 U/ml penicillin (Gibco, USA), 100 mg/ml streptomycin (Gibco, USA), 10 mM β-glycerophosphate (Sigma, USA) and 50 μg/m ascorbic acid (Sigma, USA). For osteoclastogenic induction, the cells were cultured in generic (α-MEM supplemented with 10% FBS, 100 U/ml penicillin, and 100 mg/ml streptomycin).

### Histochemistry

The treatment of hPTH(1–34) (10 nM) and GR(1–34) (10 nM) were followed a 4/48 h intermittent cycling plan [31]. In brief, the cells were cultured in the medium supplemented with peptides for 4 h and changed into the fore-mentioned medium without peptide for 44 h prior to the next treatment. At the 14th days of culture, ALP and mineralized nodules were examined by histochemical staining. The cells were fixed with 4% paraformaldehyde for 30 min, gently rinsed with PBS and stained for ALP with BCIP/NBT Alkaline Phosphatase Color Development Kit (Beyotime Institute of Biotechnology, Haimen, China), or for mineralized nodule demonstration with 1% Alizarin Red S solution (Sigma, St Louis, MO, USA) for 30 min at room temperature. ALP activity was measured by incubating cell lysates (extracted with 0.2% TritonX-100) in ALP substrate buffer containing the soluble substrate p-nitrophenyl phosphate. The quantification was performed based on the absorbance at 520 nm (Jiancheng Bioengineering Institute, Nanjing, China). To determine the calcium content of the cultures, cells were washed in Ca^2+^- and Mg^2+^-free PBS and then, incubated for 3 h in 0.2 ml of 0.6 N HCl. Extracted calcium was then measured spectrophotometrically at 610 nm after the reaction with methylthymol blue (Jiancheng Bioengineering Institute, Nanjing, China). TRAP staining was performed after the 7 days of treatment by using a TRAP staining kit. 10 microscopic fields (10 × 10) were randomly selected for the TRAP positive cells counting. The percentages of TRAP positive cells to total cells were calculated for quantification.

### Real-time PCR

Bone marrow stromal cells were isolated and plated into 6 well plates at 4 × 10^6^ cells/cm^2^ as described above. After 48 h cultured in the generic medium, cells were subjected to hPTH(1–34) (10 nM), hPTH(1–34) (10 nM) combined with 1 μmol/L U73122 (PLC inhibitor, Abcam, United Kingdom) or GR(1–34) (10 nM). Total RNA was isolated after 4 h later by using RNeasy Mini Kit (Takara BIO, Dalian, China). Expressions of Receptor Activator of Nuclear Factor κ B (RANK) and its ligand (RANKL), osteoprotegrin (OPG), nuclear factor of activated T cells (NFATC) 1, TRAP, Cathepsin K were measured by two-step real-time RT-PCR. Briefly, the first strand of cDNA was synthesized according to the manufacturer’s instructions using a PrimeScript® RT reagent Kit (Takara BIO, Dalian, China). For each gene, two specific PCR primers (RANK/fw, 5′- ACCTCCAGTCAGCAAGAAGT-3′, RANK/re, 5′-TCACAGCCCTCAGAATCCAC-3′; RANKL/fw, 5′-AGCCGAGACTACGGCAAGTA-3′, RANKL/re, 5′-GCGCTCGAAAGTACAGGAAC-3′; OPG/fw, 5′-ACCTCACCACAGAGCAGCTT-3′, OPG/re, 5’-TTGTGAAGCTGTGCAGGAAC-3′; NFATC1/fw, 5′- CCGTTGCTTCCAGAAAATAACA-3′, NFATC1/re, 5’-TGTGGGATGTGAACTCGGAA-3′; TRAP/fw, 5’-TCCTGGCTCAAAAAGCAGTT-3’;TRAP/re, 5’-ACATAGCCCACACCGTTCTC-3′; Cathepsin K/fw, 5’-CTGAAGATGCTTTCCCATATGTGGG-3′, Cathepsin K/re 5′- GCAGGCGTTGTTCTTATTCCGAGC-3′, GAPDH/fw, 5′-TGTCGTGGAGTCTACTGGTG-3′; GAPDH/re, 5′-GC ATTGCTGACAATCTTGAG-3′) were designed and synthesized by Life Technologies (Shanghai, China). The PCR reactions (94 °C, 20 s; 60 °C, 20 s; 72 °C, 20 s) were performed on an GeneAmp® PCR System 9700 (Applied Biosystems, CA, USA) using a SYBR® Premix EX Tap™ (Takara BIO, Dalian, China). Gene expression was normalized to that of GAPDH and then expressed as fold over control.

### Statistical analysis

All statistical analyses were conducted using SPSS version 13.0 (SPSS Inc., Chicago, IL). The results are presented as means ± standard error of the mean (SEM). The significance of differences in BMDs, BMCs, the values of three-point bend testing and CT between treatment groups was analyzed by analysis of variance (ANOVA) with Bonferroni’s test for post hoc analysis.

## Results

### Generation of the orchiectomized model for fracture healing

Compared to the sham-operated mice, the serum testosterone in the orchiectomized mice began to decrease from the third week on until to the end of fifth week after ORX (Additional file 1: Figure S1A). Less trebacular bone and thinner cortical bone were detected in the growth plate of the tibia at the third and fifth week after ORX (Additional file 1: Figure S1B). Although the post-surgery bone volume [BV/TV (%)] was similar to that in the sham animals at the third week after ORX (*p* > 0.05), it was reduced dramatically at the fifth week after ORX compared to the sham animals, reflecting that the loss of bone volume was associated with the decreased testosterone (Additional file 2: Figure S1C).

To address the influence of the decreased testosterone on fracture healing, 3D reconstructions of the healing fracture after 2 weeks of fracture were performed and indicated that both the bone mass and bone volume in the fracture area were significantly decreased in ORX mice (Additional file 2: Figure S2A, B), which was coincided with the slow healing and bone shape recovery in the ORX mice after 4 weeks of fracture (Additional file 2: Figure S2A). Since the fracture healing was greatly retarded in ORX mice compared to the sham group (Additional file 2: Figure S2B), the ORX mice was an ideal model to study bone repairing during androgen deficient osteoporosis.

### The stronger healing effect of GR(1–34) during the early fracture in ORX mice

**Fig. 1:**
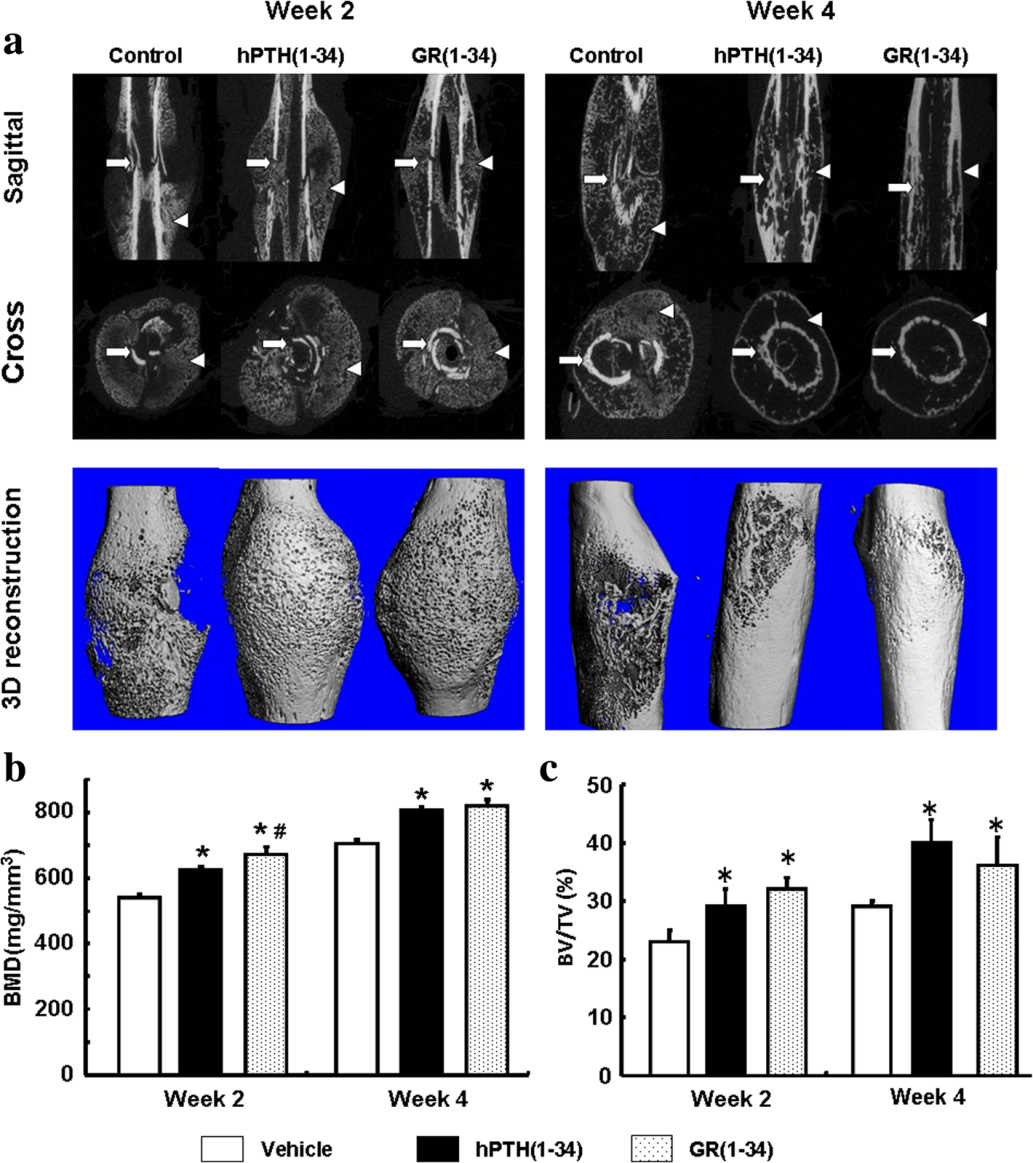
The effects of hPTH(1–34) and GR(1–34) on bone fracture healing in ORX mice. **a** The 2D and 3D images from micro-CT scanning at 2 weeks (*left panel*) and 4 weeks (*right panel*) after peptide injection showed the cortical bone (white arrows) and cancellous bone (triangles) in the fracture region. **b** The statistical analysis on BMD from micro-CT scanning. **c** The statistical analysis on bone volume [BV/TV (%)] from micro-CT scanning. [Each group contained 12 cases; *P < 0.05 for hPTH(1–34) &GR(1–34) vs. vehicle; #*P* < 0.05 for hPTH(1–34) vs. GR(1–34)]

**Fig. 2:**
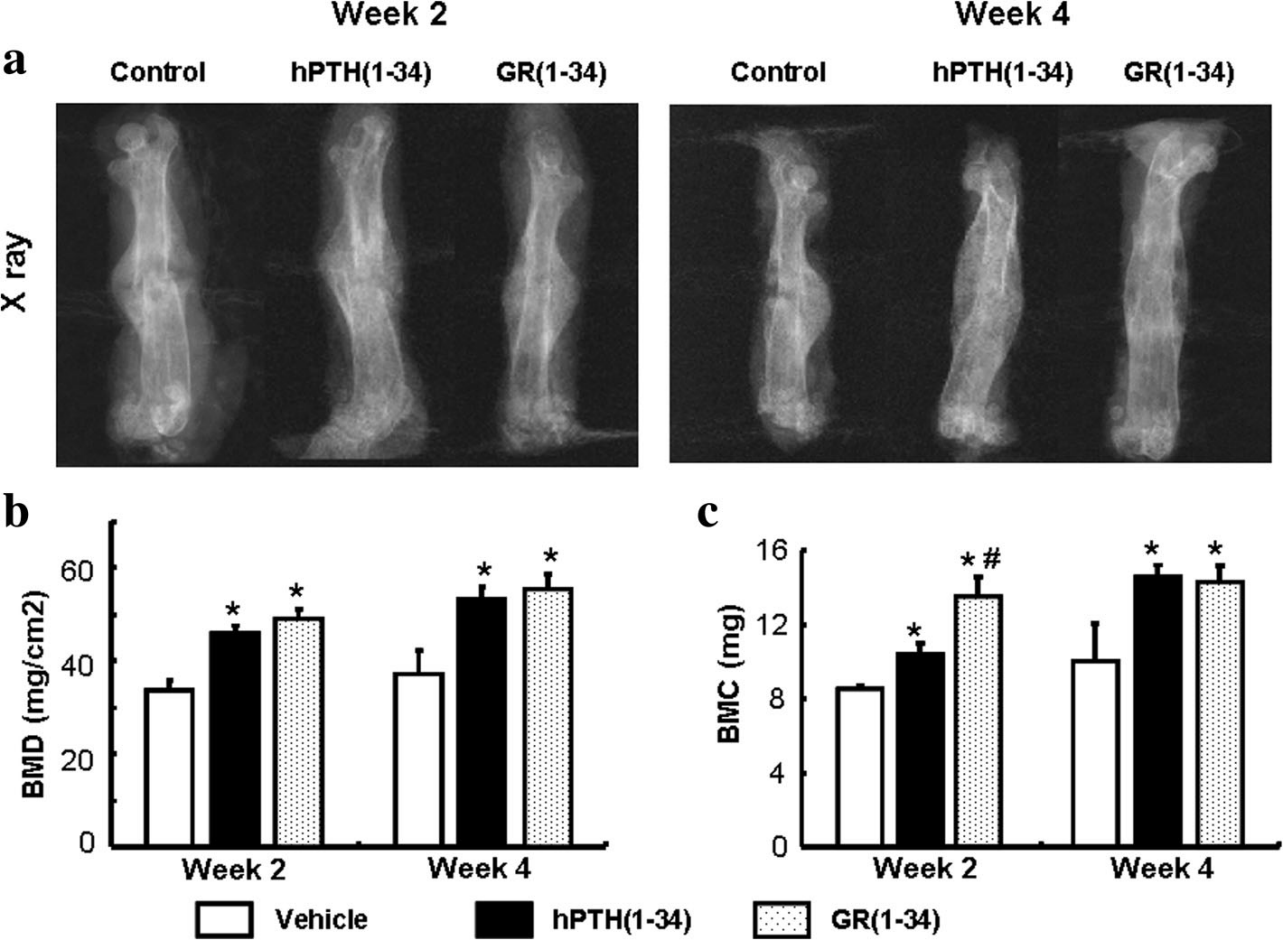
Bone mineral density and bone mass content in the fracture area measured with DEXA. **a** The plain X-ray images for femur alignment at the 2nd (*left panel*) and 4th week (*right panel*) after peptide injection. **b** The statistical analysis on BMD from DEXA. **c** The statistical analysis on bone volume [BV/TV (%)] from DEXA. [Each group contained 12 cases; *P < 0.05 for hPTH(1–34) &GR(1–34) vs. vehicle; #*P* < 0.05 for hPTH(1–34) vs. GR(1–34)]

According to the 3D BMD and BV/TV (%) from micro-CT and the 2D BMD and BMC from DEXA, the bone callus was formed in hPTH(1–34), GR(1–34) and control groups after 2 week of fracture, but there were more bone tissues in the hPTH(1–34) and GR(1–34) treated animals than that of the vehicle controls (*N* = 12 in each group; Fig. 1a, *left panel*). Micro-CT scanning revealed that after 4 weeks of fracture, the cortical bones in the ORX mice treated with hPTH(1–34) or GR(1–34) were continuously aligned and the bone callus absorbed, indicating a completely recovered bone fracture. In contrast, the cortical bone treated with vehicle control was discontinuous and the bone callus was still prominent (N = 12 in each group; Fig. 1a, *right panel*). The BMD of the fracture treated with hPTH(1–34) and GR(1–34) were significantly higher than that of mice treated with the vehicle control after two weeks of fracture (Fig. 1b). Moreover, GR(1–34) exhibited a greater enhancement on 3D BMD than hPTH(1–34) (Fig. 1b). In addition to BMD, hPTH(1–34) and GR(1–34) also significantly increased the BV/TV (%) compared to the vehicle control (Fig. 1c), though the effect on BV/TV (%) of hPTH(1–34) was similar to that of GR(1–34) (Fig. 1c). Further investigations disclosed that after 4 weeks of fracture, the increasing BMD and BV/TV (%) of the healing sites in the hPTH(1–34) and GR(1–34) groups were both evidently greater than those in vehicle treated mice (Fig. 1b). Even the difference of BMD between the hPTH(1–34) and GR(1–34) treated mice after 2 weeks of fracture was diminished after 4 weeks of fracture (Fig. 1b). Consistent with the outcomes of 3D micro-CT reconstruction, the plain X-ray images confirmed that the well aligned cortical bone in the fracture ends (Fig. 2a). DEXA confirmed that hPTH(1–34) and GR(1–34) treatments significantly increased BMD and BMC after 2 weeks of fracture (Fig. 2b), as well as the greater BMC in GR(1–34) group compared with the hPTH(1–34) group (Fig. 2c). Similarly, the BMD and BMC of hPTH(1–34) were close to those of GR(1–34) treated mice after 4 weeks of fracture (Fig. 2b, c). In summary, the treatments with either hPTH(1–34) or GR(1–34) could increase the rate of bone healing, while the GR(1–34) appeared to have a greater enhancement on the early fracture healing than hPTH(1–34).

### The biomechanical characteristics in the fractured bone of ORX mice

**Fig. 3:**
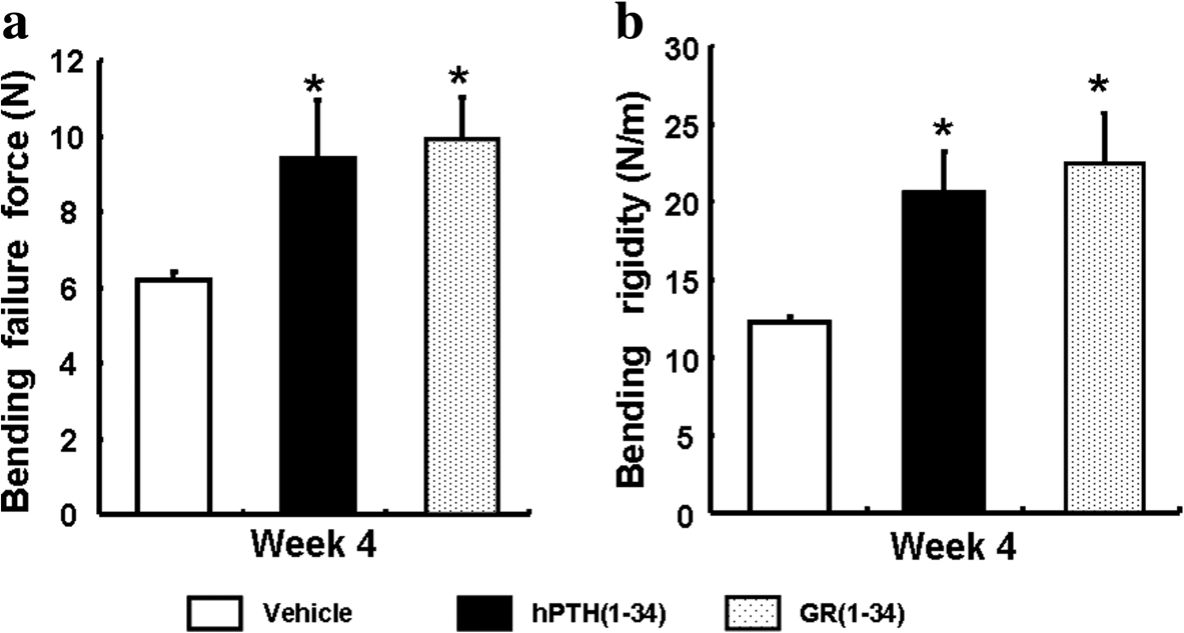
Mechanical characteristics of the fractured bone in ORX mice. After 4 weeks of peptide or vehicle administration, the bending failure force (**a**) and Bending rigidity (**b**) were measured in the ORX mice for statistical analyses. [Each group contained 6 cases; *P < 0.05 for hPTH(1–34) &GR(1–34) vs. vehicle]

Since the the bone is too soft to endure the mechanical analyses before the fourth week of fracture, the bending force and rigidity of the femurs from the hPTH(1–34) and GR(1–34) treated and vehicle control mice was compared at the fourth week after fracture. The healing femurs from both the hPTH(1–34) and GR(1–34) treated mice were able to sustain a much greater force (Fig. 3a) and exhibited an increased bending rigidity (Fig. 3b). However, there was no significant difference in the measured biomechaniccal characteristics between the femurs of mice treated with GR(1–34) and hPTH(1–34).

### The difference in bone metabolism markers between the hPTH(1–34) and GR(1–34) treated ORX mice

**Fig. 4:**
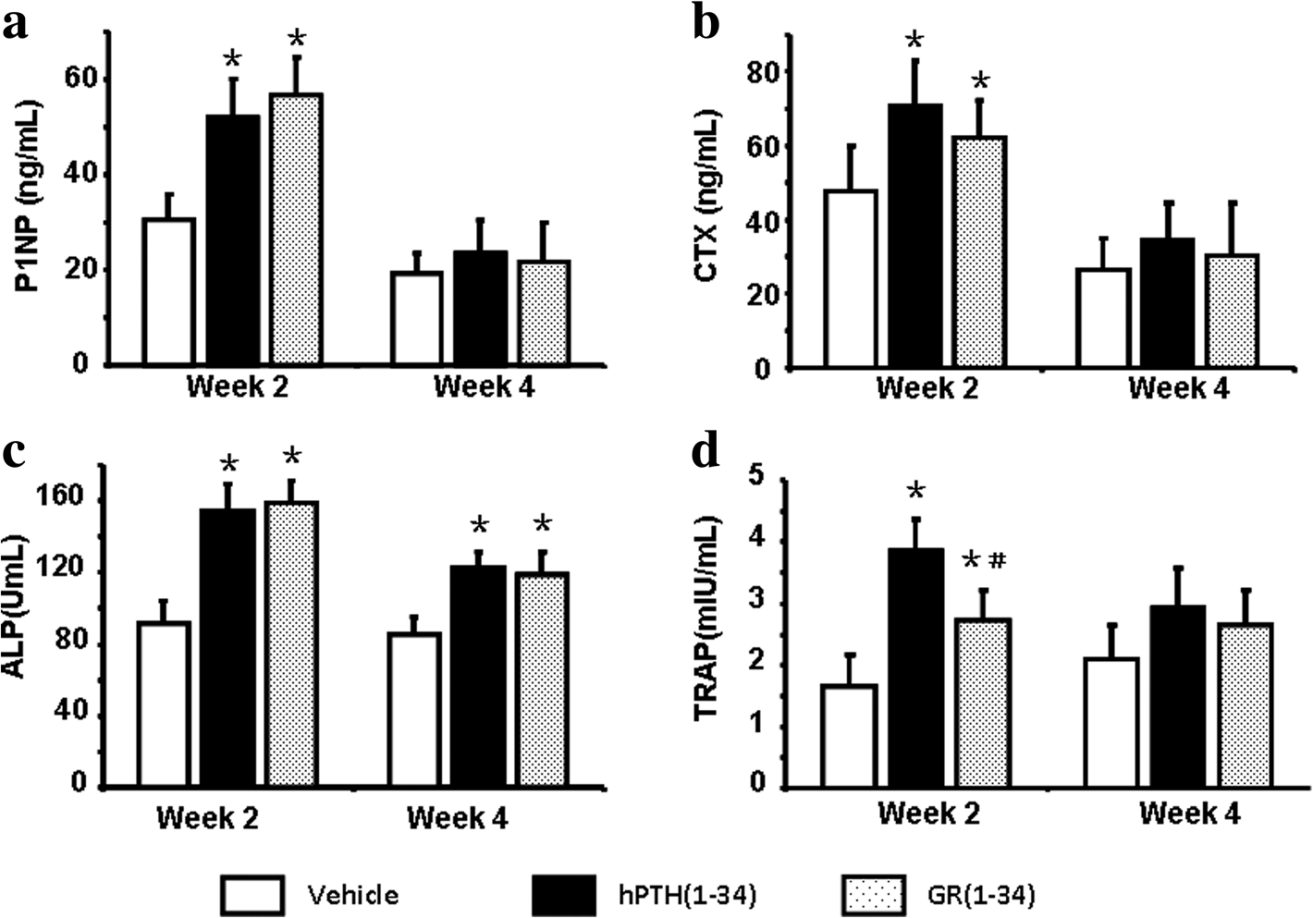
The serum levels of bone metabolic markers at 2 and 4 weeks after peptide administration in fractured ORX mice. Serum levels of P1NP (**a**), CTX (**b**), ALP (**c**) and TRAP (**d**) at the 2nd and 4th week after peptide administration were measured and present in a statistical summary. [Each group contained 12 cases; *P < 0.05 for hPTH(1–34) &GR(1–34) vs. vehicle; #*P* < 0.05 for hPTH(1–34) vs. GR(1–34)]

The serum levels of P1NP, CTX and TRAP in both the hPTH(1–34) and GR(1–34) groups remarkably increased at the second week and returned normal as control at the fourth week after fracture (Fig. 4a, b, d). Interestingly, the ALP levels in both the hPTH(1–34) and GR(1–34) groups kept higher than control from the second to the fourth week after fracture (Fig. 4c). Moreover, hPTH(1–34) only induced a higher TRAP level than GR(1–34) at the second week after fracture (Fig. 4d).

### The quicker callus transformation and osteoclastogenesis in hPTH(1–34) treated fracture healing

**Fig. 5:**
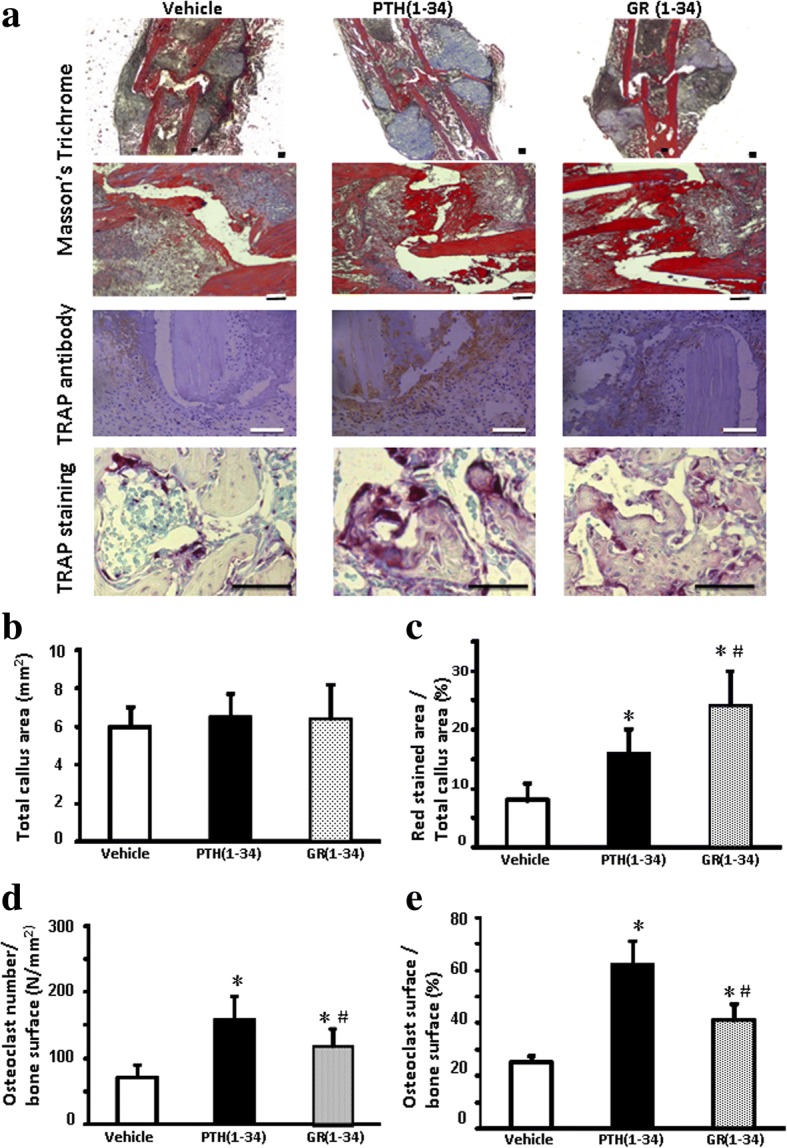
The effects of hPTH(1–34) and GR(1–34) on callus transformation and osteoclasts formation in the healing area at 2 weeks after fracture. **a** The upper two panels showed the Masson-Goldner Trichrome stain for the cartilage (in blue) and bony (in red) callus formation; the third panel showed the immunohistochemical staining with the monoclonal antibody against TRAP; the bottom panel showed the distribution of osteoclasts on the surface of bone stained by TRAP activity. Histomorphometry of callus (in blue and red) and the proportion of bony callus among total callus were statistically shown in (**b**) and (**c**), respectively. Statistical analyses were shown for the number of osteoclasts per unit area (**d**) and their surface in proportion to bone surface (**e**). [Each group contained 6 cases; scale bar represents 100 μm; *P < 0.05 for hPTH(1–34) &GR(1–34) vs. vehicle; #*P* < 0.05 for hPTH(1–34) vs. GR(1–34)]

Since GR(1–34) increased more BMD than hPTH(1–34) in the first two weeks after fracture, the histological features in the healing areas at the two weeks after fracture were analyzed to clarify the early healing process. Although no significant difference was found among the callus sizes of the three groups (Fig. 5a, b), the amounts of bony callus were significantly increased in both peptide-injecting groups, especially in the GR(1–34) mice compared with the vehicle group (Fig. 5a, c). Both the hPTH(1–34) and GR(1–34) treatments induced more osteoclasts around the woven bones, but the number and size of the osteoclasts in the hPTH(1–34) group were also more and larger than those in GR(1–34) group (Fig. 5a, d, e).

### The quicker induction of TRAP positive cells by hPTH(1–34) in the bone marrow cells of ORX mice

**Fig. 6:**
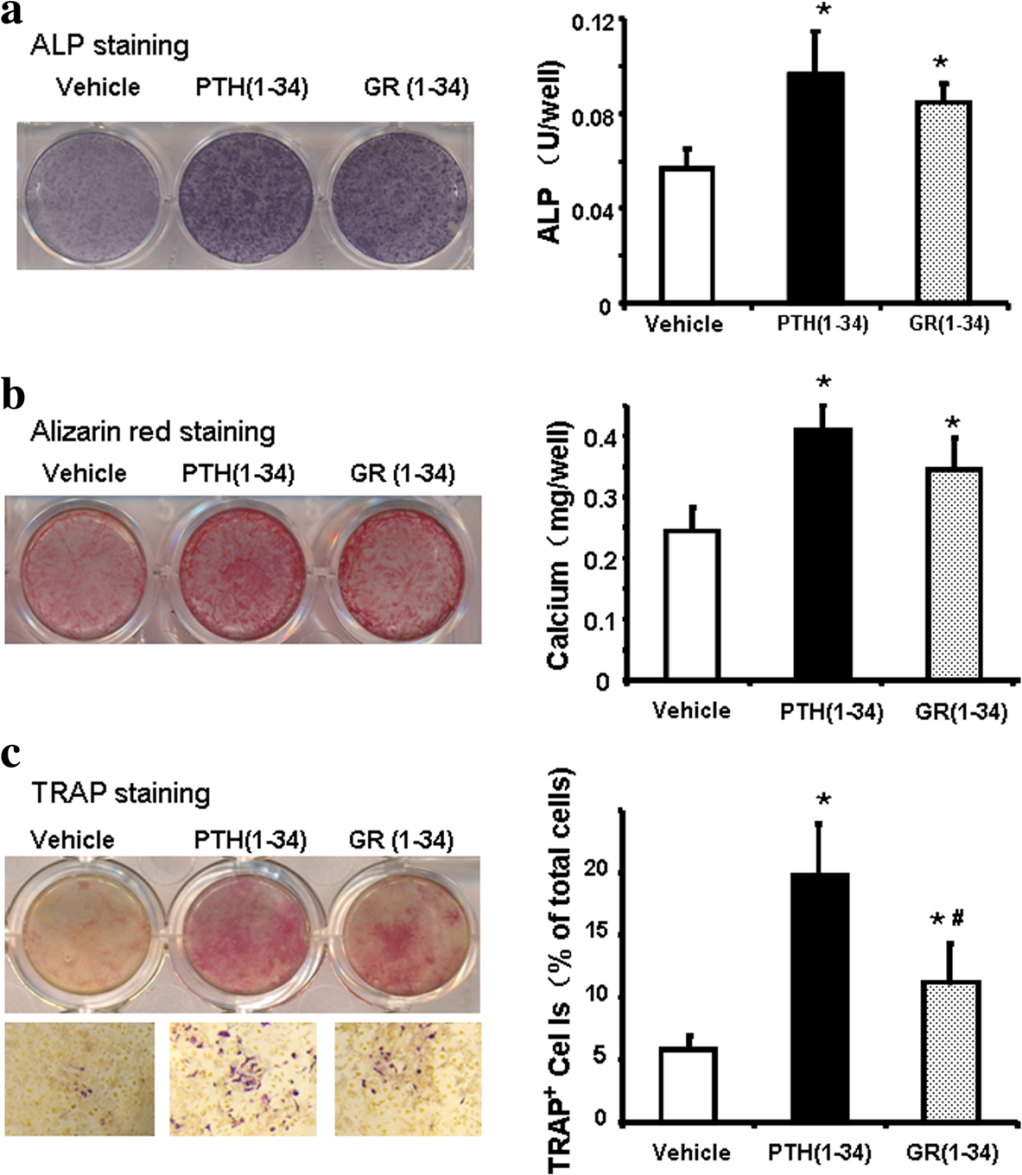
The effects of hPTH(1–34) and GR(1–34) on osteogenesis and osteoclastogenesis in bone marrow cells of ORX mice. At the 2nd week of administration, ALP activity of the cells in osteogenic medium were stained and measured with a BCIP/NBT Color Development Kit (**a**); the mineralized nodules were stained with Alizarin Red S solution and calcium deposition was quantified spectrophotometrically (**b**). At the 1st week of administration, TRAP staining was performed and the percentage of TRAP+ cells to total cells were counted (**c**). [Three independent experiments were repeated for (**a** & **b**) and six for (**c**); 10 fields at 100 magnitude were randomly selected for TRAP+ cell counting; *P < 0.05 for hPTH(1–34) &GR(1–34) vs. vehicle; #*P* < 0.05 for hPTH(1–34) vs. GR(1–34)]

The bone marrow cells isolated from the femurs of ORX mice were applied in the in vitro exploration of the soteoblastogenesis and osteoclastogenesis during the fracture healing of the androgen-deficient osteoporosis. After 2 weeks of the intermittent administration, both hPTH(1–34) and GR(1–34) could induce the ALP activity and calcium deposition at the similar intensity (Fig. 6a, b). On the other and, the intermittent treatments of both hPTH(1–34) and GR(1–34) significantly increased TRAP^+^ cells in bone marrow cells culture in 7 days. However, less TRAP^+^ cells were detected in the culture treated by GR(1–34) compared to that by hPTH(1–34). In the hPTH(1–34) group, 10.2 ± 3.4% of the TRAP^+^ cells were multinucleated, while only 2.2 ± 1.3% in GR(1–34) group (Fig. 6c). There was no multinucleated TRAP^+^ cells found in vehicle treatment.

### The quick activation on the osteoclastogenesis-associated genes by hPTH(1–34) in the bone marrow cells of ORX mice

Quantitative PCR revealed that both hPTH(1–34) and GR(1–34) administration increased the transcription of RANK, RANKL and OPG, but only the increase of RANKL expression by hPTH(1–34) was significantly higher than that by GR(1–34) in the bone marrow cells of the ORX mice (Additional file 3: Figure S3). Similarly, the hPTH(1–34) treatment was capable of inducing the robuster transcription of NFATC1, TRAP and Cathepsin K than GR(1–34) (Additional file 3: Figure S3). Surprisingly, the efficient activation of RANKL, NFATC1, TRAP and Cathepsin K expression by hPTH(1–34) could be neutralized by the PLC inhibitor U73122 (Additional file 3: Figure S3).

## Discussion

Orchiectomy has been widely used to mimic the osteoporosis in men [3]. The overall bone volume usually reduced to 40–60% of the normal within 6 to 12 weeks after orchiectomy in rats [32] and 4 weeks in mice [33], which suggested that the mice used in this study was undergoing a substantial BV loss due to androgen deprivation. Although the nadir testosterone level and its impact on mouse BV in this study were not determined, we indeed found that the weight loss of seminal vesicles and the significantly declined testosterone level after the testis removal (data not shown), which indicated the occurrence of androgen deprivation in the ORX mice. However, we acknowledged that the growth potential of the 8–10 weeks old mice might contribute to the delayed fracture healing in some extent, because the decline of testosterone was gradual and reached the half of the normal at the fifth week, instead of a rapid decline of testosterone in the previous report [34]. Even though, the differential effects of hPTH(1–34) and GR(1–34) on the fracture healing in both sham and ORX mice indicated that the consequence of the androgen deprivation in the younger ORX mice were still convincing. Besides the androgen deprivation, the lateral damages in the surrounding tissues, such as blood vessels, muscles and nerves, may also affect the fracture healing in practice. To reduce the influence of later damages on fracture healing as much as possible, the osteotomy model was adopted, as apposed of impacting bone via violence [35]. Although there were still injuries in the soft tissues during the osteotomy, their extent and variations were milder and less than those in the impacting model, which would cause mininal errors in the effects of PTH on fracture healing.

During the early stage, hPTH administration resulted mainly in the newly formed woven bones, especially the bony callus transformation, though the callus sizes were indiscriminate between the treated and untreated animals. At the 4th week after administration, the enhanced bone reconstruction was verified because of the reconnection of cortical bone and the re-absorption of cancellous bone around the fracture. Consistently, the healing bones received peptide treatment exhibited the greater mechanical characteristics than control. As previously reported, it took three weeks for the the normal mice to recover the strength and stiffness of the fractured femur [36]. However, even at the 4th week after peptide administration, the mechanical index of the fractured femur of ORX mice were still remarkably lower than that of the intact contra-lateral femurs. Therefore, we need to find out the duration required for the complete recovery from bone fracture in ORX mice in the future investigation. Interestingly, the effects of hPTH administration in ORX mice were similar to those in ovariectomized rodents [32], suggesting that the effects of hPTH is associated with sex hormones, but no discrimination between estrogen and androgen.

After 2 weeks of administration, more bone mass (BMC and BMD) and bony callus in fracture healing area of the ORX mice were induced by GR(1–34) compared with hPTH(1–34). The results of serum biochemistry suggested that compared to GR(1–34), the effect of hPTH(1–34) on bone re-absorption was much stronger. The induction of osteoclasts from the bone marrow cells verified the in vivo consequences. However, both the in vivo and the in vitro experiments revealed that the effects of hPTH(1–34) and GR(1–34) on bone formation and bone resorption in fracture healing showed no significant difference after 4 weeks of administration. Therefore, hPTH(1–34) was suggested to exert a rapid induction of osteoclasts in the fracture healing of ORX mice compared to GR(1–34).

Since GR(1–34) is the analog of hPTH(1–34) and incapable of activating PLC signaling, it is reasonable to speculate that PLC signaling contributed the rapid bone resorption during the fracture healing of ORX mice. PTH was reported to increase osteoclast formation and attachment to bone via both cAMP/PKA and PLC-coupled calcium/PKC pathways in osteoblasts [37, 38]. Although both hPTH(1–34) and GR(1–34) could increase TPAP positive mononucleated and multinucleated cells [39], our study showed that hPTH(1–34) was able to activate both mature and progenitor osteoclasts more quickly, indicated that the rapid osteoclastic commitment and maturation induced by intermittent hPTH(1–34) administration depended on PLC signaling. Since PLC (eg. PLCγ2) was reported to mediate effect of RANKL on osteoclastic differentiation [40], and the inhibitor of PLC signaling decreased the RANK expression in hPTH(1–34) treated group to that in GR(1–34) treated group, it suggested that RANKL could also be a downstream target of PLC signaling. In summary, the intermittently administrated PTH enhances fracture healing of ORX mice, while the PLC signaling activated by PTH mediates a rapid osteogenesis in the healing zone for the bone re-absorption.

Our findings on the role of PLC signaling seems controversial to the work of Guo et al. showing that the deficiency in PLC signaling significantly decreased bone volume and osteoblasts [28]. However, Guo et al. investigated the normal development, while our study focused on an androgen-associated traumatic model. Unlike the mice carrying the mutant PTH receptor, the endogenous PTH can still activate other signaling pathways in the ORX mice receiving PLC-deficient PTH analog. The differential role of PLC in bone development verses bone healing still require to be elucidated. A study on the DSEL mouse reported that DSEL mice displayed a significant decrease in the amount of trebacular bone with little alteration in the cortical bone [29], implying that PLC-dependent and -independent signaling on osteoclastogenesis were not related to PKC signaling [41]. Further study is required to reveal the roles of the PLC-dependent and -independent signaling initiated by PTH in bone resorption, which would benefit the understanding and regulating the balance of bone turnover.

## Conclusions

The PLC signaling activated by the intermittent injection of hPTH(1–34) rapidly activates the osteoclastogenesis in the fracture healing zone. This finding provides a novel insight for the effects of hPTH(1–34) on bone resorption, which would benefit the development of the new PTH analog for fracture therapy.

## Additional files

Additional file 1: **Figure S1.** Orchiectomy reduced serum testosterone and trebacular bone volume in male mice. (A) Serum testosterone levels at the third and fifth week. (B) The micro-CT scanning and 3D reconstruction of the trebacular bone of the proximal tibia. (C) Bone volume (BV/TV (%)) was measured at the third and fifth week after the surgery. (There were 12 cases in the sham and ORX groups; *p < 0.05). (TIF 478 kb)

Additional file 2: **Figure S2.** Retarded fracture healing in ORX mice. (a) The 3D reconstruction of the micro-CT scanning on the fracture region. (b) The bone volume [BV/TV(%)] was measured with micro-CT analysis at the 2nd and 4th week after fracture. (There were 12 cases in the sham and ORX groups; *P < 0.05). (TIF 762 kb)

Additional file 3: **Figure S3.** The osteoclastogenesis-associated gene expression in the bone marrow cells of ORX mice. After a hours culture with hPTH(1–34), hPTH(1–34) + U73122 and GR(1–34), mRNA was extracted from the bone marrow cells of ORX mice for real-time PCR. [Three independent experiments were repeated for each gene; variables were analyzed using analysis of variance (ANOVA) and Bonferroni’s test for post hoc analysis; *P < 0.05 for hPTH(1–34) &GR(1–34) vs. vehicle; #*P* < 0.05 for hPTH(1–34) vs. GR(1–34)]. (TIF 470 kb)

## Abbreviations

ALP: Alkaline phosphatase
BMC: Bone mass content
BMD: Bone mineral density
BV/TV: Bone volume
cAMP: cyclic adenosine monophosphate
CTX: Terminal collagen-type I fragments
DEXA: Dual-energy X-ray absorptiometry
EDTA: Ethylenediaminetetraacetic acid
GR(1–34): Ser^1^- > Gly^1^ and Glu^19^- > Arg^19^ into hPTH (1–34)
HE: Hematoxylin and eosin
hPTH: Human parathyroid hormone
Micro-CT: Micro-Computed tomography
NFATC: Nuclear factor of activated T cells
OPG: Osteoprotegrin
ORX: Orchiectomized
P1NP: Terminal propeptide of type I collagen
PKA: Protein kinase A
PKC: Protein kinase C
PLC: Phospholipase C
RANK: Receptor Activator of Nuclear Factor κ B
RANKL: Receptor Activator of Nuclear Factor κ B ligand
TRAP: Tartrated-resistant acid phsphatase
