# Supplementary figures and images for: Phospholipase C signaling activated by parathyroid hormone mediates the rapid osteoclastogenesis in the fracture healing of orchiectomized mice
Source: BMC Musculoskelet Disord. 2018 Aug 29;19:311. doi: 10.1186/s12891-018-2231-3 (PMC6116492; doi:10.1186/s12891-018-2231-3)

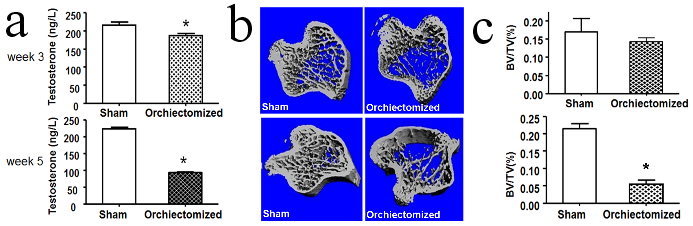

Supplement: Supplementary file 1 — Figure S1. Orchiectomy reduced serum testosterone and trebacular bone volume in male mice. (A) Serum testosterone levels at the third and fifth week. (B) The micro-CT scanning and 3D reconstruction of the trebacular bone of the proximal tibia. (C) Bone volume (BV/TV (%)) was measured at the third and fifth week after the surgery. (There were 12 cases in the sham and ORX groups; *p < 0.05). (TIF 478 kb) [file 12891_2018_2231_MOESM1_ESM.tif]

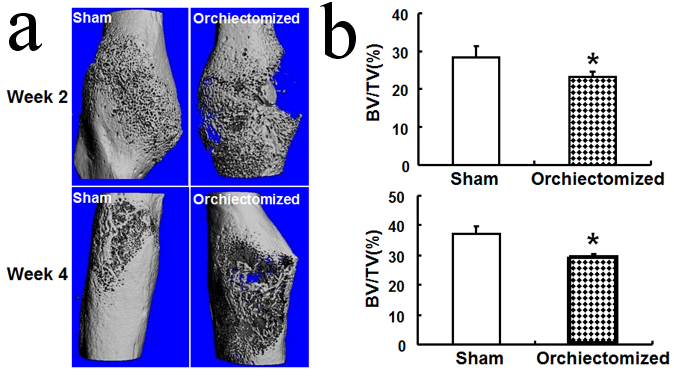

Supplement: Supplementary file 2 — Figure S2. Retarded fracture healing in ORX mice. (a) The 3D reconstruction of the micro-CT scanning on the fracture region. (b) The bone volume [BV/TV(%)] was measured with micro-CT analysis at the 2nd and 4th week after fracture. (There were 12 cases in the sham and ORX groups; *P < 0.05). (TIF 762 kb) [file 12891_2018_2231_MOESM2_ESM.tif]

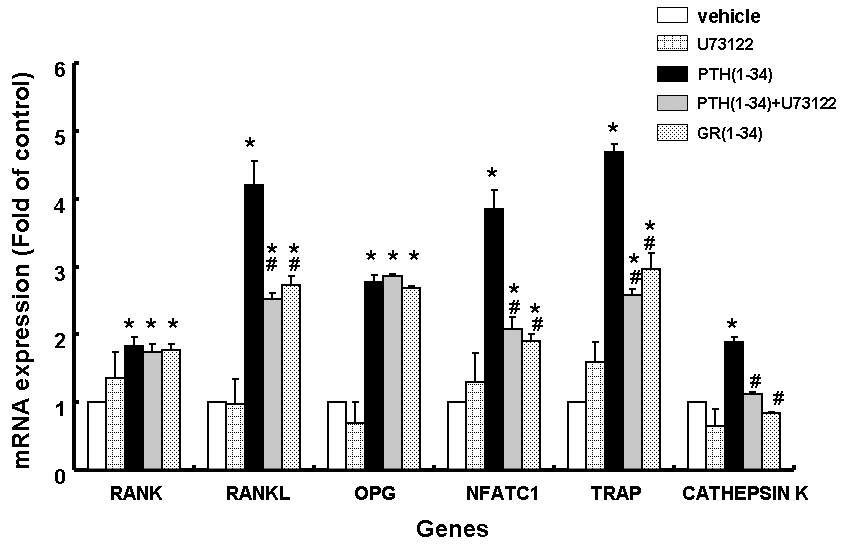

Supplement: Supplementary file 3 — Figure S3. The osteoclastogenesis-associated gene expression in the bone marrow cells of ORX mice. After a hours culture with hPTH(1–34), hPTH(1–34) + U73122 and GR(1–34), mRNA was extracted from the bone marrow cells of ORX mice for real-time PCR. [Three independent experiments were repeated for each gene; variables were analyzed using analysis of variance (ANOVA) and Bonferroni’s test for post hoc analysis; *P < 0.05 for hPTH(1–34) &GR(1–34) vs. vehicle; #P < 0.05 for hPTH(1–34) vs. GR(1–34)]. (TIF 470 kb) [file 12891_2018_2231_MOESM3_ESM.tif]
